# Systematic review of Apgar scores & cyanosis in Black, Asian, and ethnic minority infants

**DOI:** 10.1038/s41390-024-03543-3

**Published:** 2024-09-14

**Authors:** Frankie J. Fair, Amy Furness, Gina Higginbottom, Sam J. Oddie, Hora Soltani

**Affiliations:** 1 College of Health, Wellbeing and Life Sciences, Sheffield Hallam University, Sheffield, UK; 2 School of Health Sciences, University of Nottingham, Nottingham, UK; 3 Bradford Neonatology, Bradford Teaching Hospitals NHS Foundation Trust, Bradford, UK

## Abstract

**Background:**

Apgar score and cyanosis assessment may disadvantage darker-skinned babies. This review explored cyanosis and Apgar score assessments in Black, Asian, or minority ethnic neonates compared to White neonates.

**Material and methods:**

Four databases were searched. Studies of any methodology were included. A narrative synthesis was undertaken.

**Results:**

Ten studies were included. Three studies involving over 39 million neonates showed Apgar score ≤3 was predictive of neonatal mortality across all ethnicities. Black babies with Apgar score ≤3 had lower mortality rates before 28 days, however, variations in scoring practices were also observed. Three further studies (*n* = 39,290,014) associated low Apgar scores with poorer mental development up to 22 months, especially in mixed ethnicity and Black infants. One study reported inadequate training in assessing ethnic minority neonates. Cyanosis was the focus of three included studies (*n* = 455) revealing poor visual assessment of cyanosis across ethnicities. With pulse oximetry occult hypoxemia occurred slightly more frequently in Black neonates. Tongue color indicated oxygen requirement at birth, regardless of ethnicity.

**Conclusions:**

Apgar scores correlate well with neonatal mortality in all ethnicities, however scoring variations exist. Cyanosis assessment is challenging, with tongue and lips the best places to observe in the absence of pulse oximetry.

**Impact:**

- Assessment of the color component of the Apgar score and of cyanosis visually are not accurate in babies with darker skin. Small racial differences may exist for pulse oximetry in neonates, but it is more reliable than visual assessment.

## Introduction

Ethnic inequalities in maternal and neonatal healthcare provision are increasingly being recognized.^1,2^ In the United Kingdom (UK), maternal mortality is two times higher for women from an Asian background and almost four times higher among Black women than women of White ethnicity.^1^ Similarly, there are ethnic inequalities in neonatal mortality within the UK (with 2.94, 2.22, and 1.68 neonatal deaths per 1000 live births for Black, Asian and White neonates respectively) and stillbirth (with 7.52, 5.15, and 3.30 stillbirths per 1000 births for Black, Asian and White babies respectively).^2^ Awareness is increasing that Black and Asian neonates may be disadvantaged by routine neonatal assessments that are based on normative White populations.^3^ Concerns have particularly been raised over neonatal assessments that require assessment of skin color, which is subject to observer and potential racial bias.^4,5^

The Apgar score is a routine perinatal practice which includes skin color assessment. The Apgar score assesses five components: the neonate’s heart rate, respiratory effort, reflex irritability, muscle tone, and appearance.^6^ The birth attendant attributes a score of 0–2 to each of these five components at 1 and 5 min after the birth, with a maximal possible score of 10^6^. In particular, a score of 0 on the appearance component is defined as the neonate’s skin being “blue all over”, a score of 1 as “centrally pink with blue extremities”, and a score of 2 as the skin being “pink all over”.^6^ The Apgar score is extensively used across the world, as a standardized, convenient way for healthcare providers to report neonatal condition at birth.^7^ In general, an Apgar score of ≥7 indicates the neonate was born in a good condition, while a score of ≤3 is very low and indicates that the neonate was in a poor condition at birth.^7^ The reliability of the Apgar score is however questioned,^8^ particularly that the subjective assessment of skin color may disadvantage neonates with darker skin tones. To determine wellbeing more accurately in African, Caribbean, and Asian neonates’ researchers have suggested extended assessment is needed.^6^

Concerns have also been raised around the detection of neonatal hypoxia. This is typically portrayed as a neonate’s lips or skin appearing “blue” or “pale”.^3^ It is difficult to diagnose poor oxygenation in infants through visual assessment alone, with reliability varying between clinicians, including in darker skinned infants.^9^ Guidelines recommend that observation of skin color is insufficient to ascertain neonatal oxygenation.^10,11^ Pulse oximetry utilizes a non-invasive approach to assess oxygenation. Screening newborns with pulse oximetry is routinely recommended to identify heart conditions that may not be detected on visual examination.^12^ However, pulse oximeters have mainly been calibrated on White skin.^13^ Racial bias in pulse oximetry has been suggested to place Black adults at increased risk of occult hypoxemia.^14^ These inequalities in healthcare provision were particularly highlighted during the COVID-19 pandemic, where inconsistencies in technology such as pulse oximeters resulted in dark skinned individuals having a greater likelihood of inaccurate readings.^15,16^

The aim of this review was therefore to examine associations between neonatal examinations that include a subjective assessment of skin color and any objective measure of neonatal wellbeing in Black, Asian, or minority ethnic neonates compared to their White counterparts. This manuscript specifically focuses on Apgar score and the detection of cyanosis.

## Methods

This systematic review was performed according to the protocol published on PROSPERO (CRD42022344617). This manuscript considers the neonatal assessments of Apgar score and cyanosis.

### Data sources

A comprehensive electronic search was undertaken in MEDLINE, CINAHL, and PsycINFO databases. Database were searched from inception until 30th April 2022, with the search updated on 31st August 2023. Gray literature such as statutory body reports or consultation exercise reports were also searched for using OpenGrey within the DANS EASY archive. Searches included medical subject headings (MeSH) combined with key text words. These included terms around Apgar score, cyanosis, hypoxia, neonate, and ethnicity. Appendix S1 provides an example full search strategy. Only English language articles were considered.

As well as the above digital searches a group of stakeholders, including healthcare providers, academics, commissioners, and maternity user representatives, were asked via email regarding their awareness of relevant literature for inclusion.

### Eligibility criteria and study selection

Two researchers independently screened citations by title and abstract against the inclusion criteria. The inclusion criteria were any qualitative, quantitative or mixed methods study that explored detection of cyanosis or examined associations between Apgar score and an objective measure of neonatal wellbeing included but were not limited to blood gas, intensive care admission, mortality, ongoing development. Studies were included if they compared Black, Asian, or “dark” skinned neonates (less than 4 weeks old) to their White counterparts or those defined as “light” skinned within the included study. Studies undertaken in any country were eligible for inclusion regardless of development as defined by the United Nations Human Development Index.^17^ Reviews were not eligible for inclusion per se, however, references of reviews on a related topic were screened for additional primary studies, as were the references of all included studies. Two researchers independently screened all potentially relevant citations at full text. The whole research team discussed disagreements over inclusion.

### Critical appraisal

Included studies were critiqued using the Mixed Methods Appraisal Tool.^18^ This incorporates two screening questions, followed by five quality criteria relating to the appropriateness of methodology, data collection, and data analysis. These five quality criteria differ for five study design categories: qualitative, quantitative–randomized, non-randomized and descriptive, and mixed methods. Two researchers independently critiqued the included studies, with disagreements discussed with a third researcher until agreement was reached.

### Data extraction

Two reviewers undertook data extraction using a pre-defined template to record relevant information. This included; title, author, year of publication, country of origin, study design, methodology (setting, data collection, data analysis), sample size, ethnicity and age of included neonates, study outcomes according to ethnic subgroups, covariates adjusted for within analyses, source of study funding. The method used to classify ethnicity or race within each included study was noted. Where ambiguities were noted authors of the original published report were contacted for clarification.

### Data synthesis

A narrative synthesis approach^19^ was utilized, with Apgar score and Cyanosis/Hypoxia considered separately. Consideration was given to neonatal ethnicity within the included studies. Consideration was given to potentially confounding factors including socioeconomic status, education level, and maternal age.

## Results

**Fig. 1:**
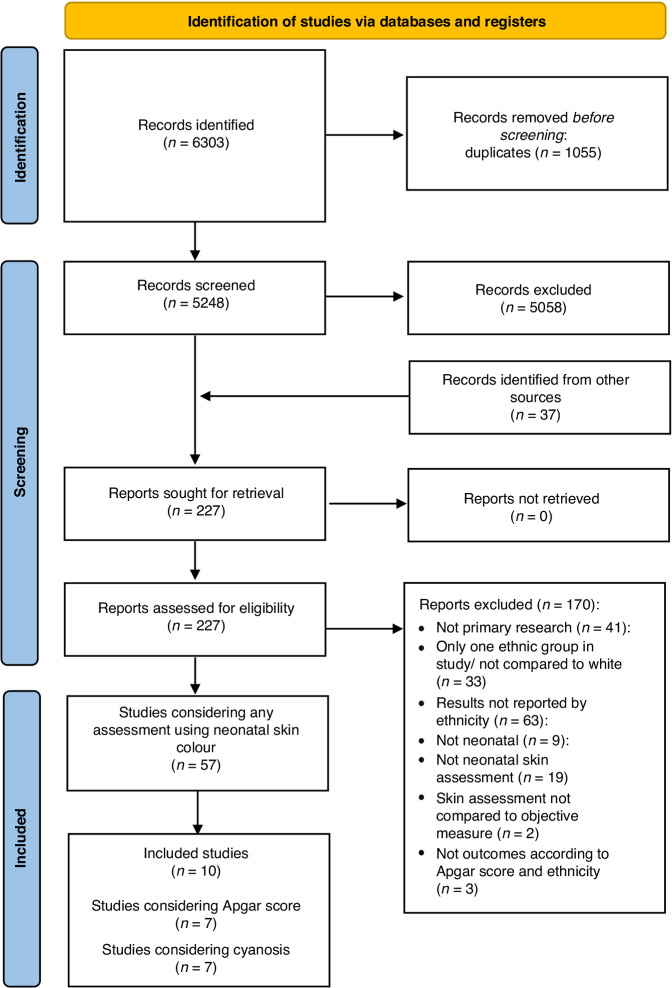
PRISMA flow chart. PRISMA flow chart showing study identification.

Of the 6303 citations obtained from database searches and the 37 studies identified through other sources, 227 were screened at full text. In total 10 studies were eligible for inclusion with seven studies considering the Apgar score^7,20–25^ and three studies considering cyanosis.^9,16,26^ Fig. 1 provides a PRISMA flow diagram of the study search and selection process.^27^ Table S1 provides detailed reasons for exclusion of full-text articles.

### Study characteristics

Study characteristics are provided in Table 1. All but one study was conducted in a county with a very high human development index (HDI),^17^ including Australia (*n* = 1), UK (*n* = 1) and United States of America (USA) (*n* = 7). The other study^21^ took place in Zimbabwe (HDI 0.593) which has a medium HDI.^17^ In total data from 39,291,376 neonates contributed to the included studies, 39,290,921 within studies considering the Apgar score and 455 in studies considering cyanosis. Table S2 reports the funding received for the included studies, with no concerns identified.

**Table 1:** Characteristics of included studies.

Author and country	Aim	Sample size	Neonatal characteristics	Ethnicity details	Methodology	Limitations	Study Conclusion
**Apgar score study characteristics**							
Chubb et al.^24^ (UK)	To review training, to assess Black, Asian, and minority ethnic babies	67 midwives and students on placement	NA	NR	Study design: Descriptive	Not able to assess long term impact of training. Not all staff trained, but high turnout at 79%	The training was well evaluated but more training and research is required to improve safety of Black, Asian, and ethnic minority families
					Inclusion criteria: 67/85 midwives and students at Yeovil District NHS Foundation Trust who completed training		
					Data Collection: Anonymous pre and post training surveys		
					Analysis: descriptive statistics and thematic analysis of qualitative data		
Gillette et al.^25^ (USA)	To evaluate the association between maternal race, 5-min Apgar score, and mortality	6,809,653 infants	Normal Apgar score (7–10) 98.8%; Intermediate Apgar score (4–6) 0.9% and low Apgar score (0–3) 0.3%	Non-Hispanic White—52.8%	Study design: Population based cohort study	Missing data within the data set for some variables as the study used routinely collected data. Maternal race only was used as there was a high proportion of missing data for paternal race.	Low Apgar scores are poorer at discriminating risk of mortality in Black and non-Hispanic non- Asian neonates than White infants
				Hispanic—23.7%	Inclusion criteria: Singleton infants, 37 + 0–44 + 6 weeks gestation, without congenital malformations, born to mothers over 15 years of age		
			Infant gender: Female 49.1%	Non-Hispanic Black—13.8%	Data Collection: From linked live birth and infant death datasets.		
				Non-Hispanic Asian—6.6%	Analysis: Logistic regression adjusted for GA, sex, maternal BMI, education, age, parity, and smoking status and stratified by race		
				Non-Hispanic other—3.1%			
				According to self-reported maternal race			
Li et al.^7^ (USA)	To evaluate if 5-min Apgar remains pertinent in practice and to assess the Apgar in predicting infant survival	25,168,052 singletons	24–25 wks 0.18%;	Non-Hispanic White 18,095,334 (66.4%)	Study design: Retrospective linked data	Infants born in very severe condition may have been reported as a stillbirth. Proportion of each Apgar score for each ethnicity NR	The Apgar score predicts neonatal and post-neonatal adverse outcomes in term and preterm infants, and is applicable to twins and in various race/ethnic groups
		768,305 twins	26–27 wks 0.25%;	Non-Hispanic Black 4,540,838 (16.6%)	Exclusion criteria: Triplets or higher (70,387); BW <500 g or NK (84,177); GA < 24 wks or >44 wks (490,214), 5-min Apgar 0, >10 or NR (8,637,941).		
			28–29 wks 0.34%;	Hispanic 3,300,185 (12.1%)	Data Collection: From linked live birth and infant death datasets.		
			30–31 wks 0.56%;	Other 1,334,801 (4.9%)	Analysis: SAS v9.2. Chi-Square, Kaplan-Meier curve, Cox proportional hazard-model adjusted for maternal education, marital status, time AN care started, and smoking during pregnancy. Non-Hispanic White referent		
			32–33 wks 1.13%;	According to maternal race			
			34–36 wks 7.38%;				
			37–41 wks 86.95%;				
			41–44 wks 3.22%				
Mihoko Doyle, et al.^22^ (USA)	To assess the predictive validity of 1-min Apgar scores on infant mortality looking at race/ethnic-specific variation.	6,544,004 neonates	White: <1500 g 0.6%; ≥1550 g & GA ≤ 36 wks 6.7%; ≥1550 g & GA > 36 wks 92.7%	5,100,942 White, 1,252,870 Blacks, 190,192 MA	Study design: Retrospective linked data	Apgar score involves some subjectivity. Relatively small number of Mexican Americans included in study	Apgar is a strong predictor of infant survival including within race/ethnic groups
			Black: <1500 g 2.0%; ≥1550 g & GA ≤;36 wks 15.5%; ≥1550 g & GA > 36 wks 82.5%	NR how attributed ethnicity/race	Exclusion criteria: Multiple births; BW <500 g; <22 wks GA		
			MA: <1500 g 0.7%; ≥1550 g & GA ≤ 36 wks 8.8%; ≥1550 g & GA > 36 wks 90.5%		Data Collection: 1989–1991 NCHS Linked Birth/Infant Death files for the USA 1-min Apgar scores. Classification as BW<1500 g, or >1500 g with GA ≤ 36 wks or GA>36 wks		
			Apgar (0–3): White 1.7%, Black 3.1%, MA 1.8%; Apgar (4–6): White 5.9%, Black 7.3%, MA 5.8%; Apgar (7–10): White 92.4%, Black 89.6%, MA 92.4%		Analysis: Logistic regression models, with race/ethnic models estimated		
Serunian & Broman^20^ (USA)	To investigate relationship of 1-min Apgar scores to 8-month Bayley mental and motor score	350 / 391 children had Apgar and Bayley scores	1-min Apgar:	86 White, 89 Black, 75 mixed (Black & Portuguese)	Study design: Prospective cohort	NR	Children with abnormal development at 8 months had significantly lower 1 min Apgar scores
			White 0–3 (3.5%); 4–6 (16.3%); 7–10 (80.2%)	Racial designation based on maternal report	Inclusion criteria: Participants selected from the Providence Child Development Study. Selected according to race/ethnicity		
			Black 0–3 (9.0%); 4–6 (14.6%); 7–10 (76.4%)		Data Collection: 1 min Apgar score. Bayley mental and motor development form at 8-months, as well as Infant Behavior Profile and presence or absence of physical abnormalities.		
			Mixed (Black & Portuguese) 0–3 (6.7%); 4–6 (9.3%); 7–10 (84.0%)		Analysis: Chi square tests		
Shankaran et al.^23^ (USA)	To evaluate neurodevelop-mental sequelae among extremely high-risk infants (low birthweight, preterm and low Apgar)	304 infants survived to hospital discharge of whom 246 were seen at 18–22 months	GA: 23.6 ± 0.7 wks	Initial cohort Black: 182/304 (59.9%)	Study design: Prospective cohort	Not all surviving infants followed-up. Loss to follow-up may result in serious ascertainment bias, although clinical and demographic characteristics of those not evaluated were similar	High-risk infants (low birthweight, preterm, and low Apgar) are at high risk of morbidity and mortality
			BW (mean): 639.3 ± 63.6 g	Followed up 146/246 (59.3%)	Inclusion criteria: 304 infants surviving to hospital discharge from 12 NNUs participating in previous study	A center-based study therefore potential for referral bias	
			5-min Apgar <3: 24.4%	NR how attributed ethnicity/race	Data Collection: Follow up at 18–22 months corrected age. Amiel-Tison neurologic examination, Bayley Mental Developmental Index and Psychomotor Developmental Index; medical and social history including parental education, occupation, household composition, income level		
			Maternal age: 26.7 ± 6.9 yrs		Analysis: Mantel-Haenszel odds ratios		
			Infant gender: Male 44.7%				
Wolf et al.^21^ (Zimbabwe)	To document neurological condition of African neonates with a low Apgar score	165 babies in Zimbabwe compared to asphyxiated infants in the Netherlands (*n* = 94) and Grenada, Caribbean (*n* = 11)	In Zimbabwe: Primiparous 51.5%; Infant gender girl 43.6%; preterm 15.7%; congenital malformations 7%, SGA 12%	Only country of origin given:	Study design: Descriptive studies x3	Control groups not strictly comparable due to different Apgar cut off points in the different countries.	Neonatal morbidity was higher in Zimbabwe than in the comparison groups from the Netherlands and Caribbean
			BW: Zimbabwe 2846 ± 702 g; Netherlands 2879 ± 978 g.	Zimbabwe; Netherlands or Grenada - Caribbean.	Inclusion criteria: Babies with an Apgar score of ≤5 at 5 min admitted to NNU between 1 July 1991 and 30 June 1992	Unable to ascertain impact of ethnicity vs different care practices between countries	
			GA: Zimbabwe 38.5 ± 2.9 wks; Netherlands 38.4 ± 3.6 wks.		Data Collection: Comprehensive neurological examination adapted from Prechtl when sufficiently stable to tolerate the examination, but not before the third day after term delivery and when reached corrected term age if born preterm. The two reference groups defined asphyxia as an Apgar ≤ 6 at 3 min. Examinations performed by the same investigator.		
			Age mother: Zimbabwe 24 ± 6.2 yrs; Netherlands 25 ± 4.7 yrs.		Analysis: Chi square tests, Pearson’s r and ANOVA.		
Cyanosis study characteristics							
Dawson et al.^26^ (Australia)	To explore whether the pinkness of an infant’s tongue provided a useful indication that supplemental oxygen was required when pulse oximetry is unavailable	68 neonates (271 paired assessment)	GA: 38 ± 2 wks	Results given by Caucasian/non-Caucasian, proportions in each group not given.	Study design: Prospective observational study	Pulse oximeters not accurate when arterial O_2_ < 70%, so impacted by neonate low O_2_ sats in first few mins of life.	Tongue color was a specific but insensitive sign that SpO_2_ was <70%. When the tongue was pink, it was likely the infant had SpO_2_ > 70% and supplemental oxygen not required
			Mean BW: 3214 ± 545 g		Inclusion criteria: Convenience sample of infants delivered by CS between Aug and Nov 2012. Excluded if GA < 28 wks, parents did not speak English or pulse oximeter alarm messages.	Only studied infants born by CS	
			Female *n* = 36 (53%)		Data Collection: Simultaneous SpO_2_ and visual assessment of tongue color when pulse oximeter applied and at 2,3,4,5,6,7 and 10 min after birth. 38 midwives & 7 pediatric trainees carried out assessments		
			Type of anesthesia: general (*n* = 2); epidural/spinal (*n* = 66)		Analysis: Sensitivity, specificity, PPV, NPV, and positive and negative likelihood ratios of tongue not pink to detect SpO_2_ < 70%. AUC calculated.		
Goldman et al. (1973)^9^	To determine the relationship of clinical assessment of skin and mucous membrane color of neonates to arterial oxygen saturation	93 neonates (182 instances of assessment)	Birthweight: <1500 *n* = 20; 1500–2500 *n* = 52; >2500 g *n* = 21	Skin pigmentation assessed by 3 point scale - fair/medium/dark. Repeated if the infant was re-studied as pigmentation can darken in neonatal period.	Study design: Prospective cohort	NR	Trunk and ears were the least sensitive areas and hands, nailbeds, and around the mouth the most sensitive areas to detect cyanosis
			29 were ill - respiratory distress syndrome (*n* = 21), pneumonia (*n* = 1), massive aspiration (*n* = 2), congenital heart disease (*n* = 3), seizures after birth anoxia (*n* = 2). The rest were “well”	Dark skinned infants (as judged by at least 2 of the 3 observers) *n* = 27	Inclusion criteria: Infants PN age <2 wks. Excluded if axillary temp <36 °C, bruising or ecchymoses to trunk, face or hands, or if Hb <13 or >23 gm/dl.		
					Data Collection: Simultaneous clinical assessment and determination of arterial pO_2_ and pH. Assessments by 2 physicians & a nurse in brightly lit room. Cyanosis judged present or absent in 6 areas: lips, ears, trunk, nailbed, hands, region around the mouth. Blood taken after observation.		
					Analysis: Chi square with Yates correction.		
Vesoulis et al.^16^ (USA)	To determine whether oxygen saturation is overestimated using pulse oximetry for Black patients.	294 infants	Mean GA: 25.8 ± 2.1 wks (Black 25.6 ± 1.9 wks vs White 25.9 ± 2.1 wks *p* = 0.09)	124 Black (42%), 170 White (58%)	Study design: Retrospective cohort study	Syringe examined for air prior to analysis.	Modest but consistent difference in SpO_2_ error between Black and White infants, with increased incidence of occult hypoxemia in Black infants
			Mean BW: 845 ± 265 grams, (Black 805 ± 260 g vs White 875 ± 268 g *p* = 0.02.)	Classified according to parental identification on birth certificates.	Inclusion criteria: Preterm infants with GA < 32 wks, BW < 1500 g, and admitted to NNU between 2012 and 2019 with valid vital sign data and at least one arterial blood gas.	SpO_2_ sensor is rotated every 12 h, but position not routinely charted. Positions other than right upper extremity are post-ductal with potential mismatch between pre- & post-ductal measurements.	
			Black Female: 64 (52%)		Data Collection: Pulse oximetry with simultaneous arterial blood gas. During the study period alarm limits were set between 88 and 96% until infant reached 35 wks postmenstrual age, then alarm set to 88%. SpO_2_ data 30 s before to 30 s after arterial blood gas averaged	Binary race classification used based on self-reported race, further differentiation of skin tone /melanin content would be beneficial in future studies.	
			White female: 74 (44%)		Analysis: R statistical package. Univariate comparison using nonparametric methods (Fisher’s Exact or Mann–Whitney U test). Pearson correlation coefficient, linear regression, and non-linear regression		
			Received AN steroids: Black 70%; White 74% (*p* = 0.49)				

*USA* United States of America. *UK* United Kingdom. *NA* not applicable. *NR* not reported. *GA* gestational age. *BW* birthweight. *Wks* weeks. *AN* antenatal. *MA* Mexican American. *Hb* hemoglobin. *Yrs* years. *SGA* small for gestational age. *CS* Cesarean section. *AUC* Area under the curve. *PPV* positive predictive value. *NPV* negative predictive value. *NNU* neonatal unit. *Min* minute. *SpO*_*2*_ oxygen saturation using pulse oximetry.

Of the nine studies that recruited neonates, three^7,20,25^ assigned neonatal race according to maternal self-reported race and one^16^ by parental identification on the neonate’s birth certificate. One study^9^ assigned neonates as “dark skinned” if at least two of the three professionals observing the neonate judged it to be “dark skinned” and one study was undertaken across three countries and assigned neonates according to their country of origin.^21^ The remaining three studies^22,23,26^ did not stipulate how neonatal race was attributed.

### Study quality

Table 2 summarizes critical appraisal ratings for the included studies. The research question was deemed to be clear within all studies, and all but one study^21^ was deemed to have collected data that addressed the research question. Two of the seven Apgar score studies^7,25^ and none of the three cyanosis studies were assessed as low risk of bias across all five domains.

**Table 2:** Results of the critical appraisal.

Author	Clear research question	Data collected addressed research question		Quantitative non-randomized study					Quantitative descriptive study				
				Q1	Q2	Q3	Q4	Q5	Q1	Q2	Q3	Q4	Q5
Apgar studies													
Chubb et al. ^24^	✓	✓							✓	?	?	X	✓
Gillette et al. ^25^	✓	✓		✓	✓	✓	✓	✓					
Li et al. ^7^	✓	✓		✓	✓	✓	✓	✓					
Mihoko Doyle et al. ^22^	✓	✓		✓	✓	✓	?	✓					
Serunian & Broman ^20^	✓	✓		✓	✓	?	✓	✓					
Shankaran et al. ^23^	✓	✓		?	✓	✓	✓	✓					
Wolf et al. ^21^	✓	?							✓	✓	X	✓	X
Cyanosis studies													
Dawson et al. ^26^	✓	✓							✓	?	✓	?	✓
Goldman et al. ^9^	✓	✓		?	✓	✓	✓	✓					
Vesoulis et al. ^16^	✓	✓		✓	✓	X	X	✓					

✓ - adequately addressed

X – not adequately addressed

? unclear risk of bias

Quantitative non-randomized study questions:

Q1 - Are the participants representative of the target population?

Q2 - Are measurements appropriate regarding both the outcome and intervention (or exposure)?

Q3 - Are there complete outcome data?

Q4 - Are the confounders accounted for in the design and analysis?

Q5 - During the study period, is the intervention administered (or exposure occurred) as intended?

Quantitative descriptive study questions:

Q1 - Is the sampling strategy relevant to address the research question?

Q2 - Is the sample representative of the target population?

Q3 - Are the measurements appropriate?

Q4 - Is the risk of nonresponse bias low?

Q5 - Is the statistical analysis appropriate to answer the research question?

### Apgar score

Seven included studies considered the Apgar score. One of these studies considered healthcare provider training (*n* = 67). The remaining six studies compared an objective measure of neonatal wellbeing according to Apgar score among White infants and at least one group of infants with any other skin tone (*n* = 39,290,921). Of these three studies considered mortality and three considered longer term development.

Three large studies (*n* = 39,290,014) all linked birth-death datasets to consider neonatal mortality according to Apgar score and ethnicity. The studies included 6,544,004 neonates,^22^ 25,936,357 neonates^7^ and 6,809,653 neonates^25^ respectively. In all three studies, a low Apgar score (of 3 or less) was associated with increased risk of neonatal death across all ethnicities. The first study^22^ adjusted for maternal sociodemographic and health risk factors and birthweight. Compared to neonates with a 1-min Apgar score of 7–10 those with an Apgar score of 0 to 3 had lowest odds of neonatal death if they were non-Hispanic Black (OR 20.40), with White (OR 36.21) and Mexican American neonates (OR 44.24) having increased odds of neonatal death with low Apgar score (score 0–3).^22^ The same race/ethnic variation was seen for medium 1-min scores (Apgar score 4–6). However, more Black neonates received a 1-min Apgar score of 6 or lower (10.4%), compared to of White or Mexican American neonates (7.6%).^22^ The second study^7^ which was low risk of bias across all domains found at the same 5-min Apgar score neonatal mortality was consistently lower in non-Hispanic Black than non-Hispanic White neonates. The biggest difference was noted at the lowest 5-min Apgar scores (scores 1–3).^7^ This racial variation was evident for both term and preterm births. The lower risk of mortality in Black neonates with each Apgar score remained after adjusting for potential confounders including maternal smoking, education, marital status, and gestation antenatal care commenced.^7^ However, the proportion of Black neonates compared to White or Mexican American neonates receiving each 5-min Apgar score was not reported, which could have influenced these findings.^7^ The final study^25^ was also low risk of bias across all domains. They found that compared to White neonates, Non-Hispanic Black and “Non-Hispanic other” neonates with a 5-min Apgar score of 0–3 had lower early neonatal mortality (up to 6 days of age) [Black: 45.8 per 1000 (95% CI 39.5–53.0); “Non-Hispanic other”: 58.7 per 1000 (95% CI 43.7–79.1); White: 63.6 per 1000 (95% CI 58.8–68.9) respectively] and overall neonatal mortality (up to 27 days of age) [Black: 53.9 per 1000 (95% CI 43.7–61.5); “Non-Hispanic other”: 65.9 per 1000 (95% CI 49.8–87.1); White: 72.4 per 1000 (95% CI 67.2–78.04) respectively].^25^ However, the incidence of a low and intermediate Apgar score again varied by ethnicity, with low scores (Apgar score 0–3) being significantly higher in non-Hispanic Black (0.42%, *n* = 3931) and “non-Hispanic other” neonates (0.33%, *n* = 698) compared to White neonates (0.25%, *n* = 8,863) (*p* < 0.001) and intermediate scores (Apgar score 4–6) also being significantly higher in Black (1.26%, *n* = 11,816) and “non-Hispanic other” neonates (1.16%, *n* = 2464) compared to White neonates (1.02%, *n* = 36,144) (*p* < 0.001).^25^

Three studies with small sample sizes (246,^23^ 270^21^ and 391 neonates^20^ respectively) and all with concerns regarding risk of bias, explored long term development according to Apgar score for neonates of different ethnicities. The long-term predictive value of the Apgar score according to ethnicity was inconsistent within the studies. Infants with 1-min Apgar scores of 0–3 had significantly lower Bayley mental development scores (74.7 ± 12.7) and psychomotor development scores (31.4 ± 6.6) that infants with an Apgar score of 7 to 10 (Bayley mental development score 80.5 ± 3.8 and motor development score 34.5 ± 4.1).^20^ When considering ethnicity, one study found Bayley mental and psychomotor development scores at 8 months and 1-min Apgar score were only significantly correlated in those of mixed ethnicity (a group mainly consisting of mixed Portuguese and Black African descent) (*p* < 0.01 for both Bayley mental and psychomotor development scores) but not among Black only or White only infants.^20^ A separate study of infants with a 1-min Apgar score of 3 or below and low birthweight (≤750 g) similarly found that while Black race was predictive of abnormal mental development at 18–22 corrected months (OR 2.2, 95% CI 1.2–3.7) within univariate analysis, this was no longer the case after adjusting for confounders such as income and education (OR 1.9, 95% CI 0.9–3.8).^23^ Furthermore, there was no association between psychomotor development and Black race within the univariate or multivariate analysis (OR 1.2, 95% CI 0.6–2.5).^23^ The final study, exploring long-term outcomes in infants with a 5-min Apgar score <5, found more abnormal neurological classifications in infants born in Zimbabwe (35.8%, *n* = 59) than infants born in the Netherlands (0%) or the Caribbean (19.2%, *n* = 18).^21^ However, the multiple confounders within this study such as different maternal and neonatal care practices in each country meant the impact of race per se could not be ascertained.

One study that looked at healthcare professionals training on assessing Black, Asian and minority ethnic neonates undertook pre- and post-training surveys (*n* = 67).^24^ Only 9.1% (5/55) of professionals reported previously receiving specific training around care of Black and minority ethnic mothers and babies.^24^ They instead relied on self-directed learning and discussions with colleagues. Black mannequins were more likely to have been used in the education of midwives who trained in the previous 5 years (44%) than in those trained 5–10 years ago (18%). The Apgar score was felt not to be the most appropriate way to determine neonatal condition at birth by 96% of midwives after the training, due to the inappropriateness of the term “pink” for many neonates. Overall, 98% of midwives intended to make alterations to their clinical practice because of the new knowledge they had acquired during the training, with midwives describing being shocked by the impact of implicit bias resulting in inequality and inequity within maternity care.^24^

### Detection of cyanosis or hypoxia

Three studies (*n* = 455), all with concerns over risk of bias, considered the detection of cyanosis in neonates with darker skin tones compared to White neonates.^9,16,26^

The first study, of 93 participants, determined how accurately arterial oxygen saturation was predicted by visual assessment of skin color at different body sites.^9^ Skin color was a crude guide to arterial oxygen saturation in neonates regardless of skin tone.^9^ False positive observations, where professionals thought the neonate was cyanosed when arterial oxygen saturation was actually over 90%, were common when observing the hands (46%), nailbeds (57%), and around the mouth (73%). However, there were few false negatives at these sites, with cyanosis observed in the hands, nailbeds, and around the mouth in all instances where arterial oxygen saturation was below 75%.^9^ The most reliable site to detect cyanosis was the lips however this was still poor with 28% false positives, and over 25% false negatives when arterial saturations were between 80 and 89%. Neonates were classified as dark skinned if a minimum of two out of the three observers thought they had dark skin.^9^ Dark skinned neonates when compared to the overall group had fewer false positives when assessing the hands (18% compared to 46%), trunk (8% compared to 19%) or around the mouth (60% compared to 73%).^9^

The second small study of 68 neonates explored whether supplemental oxygen requirement in the first 10 min after birth could be determined by tongue color.^26^ A pink tongue generally indicated that supplemental oxygen was not required as neonatal oxygen saturation was above 70%, which was the level at which the country specific guidelines advised administration of oxygen^26^ given healthy term neonates typically take between 5 and 10 min to achieve oxygen saturations of 90%.^28^ The area under the Receiver Operator Characteristics Curve was not affected by ethnicity. Area under the curve was 0.89 (95% CI 0.84–0.95) and 0.94 (95% CI 0.87–1.00) respectively for White and “non-White” neonates.^26^ While the exact sample size of ‘non-White’ neonates was not provided, the study demonstrated that evaluating tongue color in “non-White” neonates was not less effective at detecting hypoxemia than in White neonates.

The final study examined the impact of ethnicity on pulse oximetry among 294 neonates admitted to neonatal intensive care.^16^ Overestimation of arterial saturation from pulse oximetry was 2.4-fold greater in Black than White neonates (mean bias 1.73% compared to 0.72%, *p* < 0.01). Black neonates consistently had higher pulse oximetry saturation readings at each arterial oxygen saturation level that White neonates. While the exact difference varied by pulse oximetry saturation (SpO_2_), the degree of error widened between Black and White neonates for SpO_2_ ≤ 95%.^16^ Occult hypoxemia (defined as SpO_2_ ≥ 90% with arterial oxygen saturation <85%) occurred in 9.2% of Black neonates (188/2044) compared to 7.7% of White neonates (181/2343), although the difference was not statistically significant (*p* = 0.08).^16^ The sensitivity of the pulse oximeter to detect true hypoxia (SpO_2_ < 90% when SaO_2_ < 85%) was similar for Black and White neonates (39% and 38% respectively) with specificity also similar (81% vs 78% respectively).^16^

## Discussion

### Main findings

This systematic review included 10 observational studies that considered cyanosis or Apgar score assessment and their association with neonatal wellbeing in Black, Asian, or ethnic minority neonates compared to White neonates. Three studies showed Black neonates have lower neonatal mortality rates at low Apgar score than their White counterparts.^7,22,25^ However, they were also more likely to receive a low Apgar score. Detection of cyanosis was poor for all ethnicities, with the tongue and lips the best places to observe. When using pulse oximetry, occult hypoxia was more likely in Black neonates although this did not reach significance.^16^ Only 9.1% of staff in one survey reported receiving adequate training around caring for ethnic minority babies.^24^

### Comparison with other studies

The Apgar score provides a rapid scoring of a neonate,^29^ however many of the individual elements are recognized as subjective.^6^ In particular, neonatal skin color assessment within the appearance component has been questioned, as it is least correlated with cord pH, arterial carbon dioxide and base excess.^30^

The included studies all showed mortality increased with lower Apgar scores.^7,22,25^ Black neonates had lower odds of mortality with a low Apgar score than White neonates after adjusting for multiple confounders including socioeconomic factors and maternal lifestyle.^7,22,25^ This was despite Black neonates having higher overall rates of neonatal mortality within two of the studies.^22,25^ This may partly be explained by inconsistencies in Apgar scoring according to ethnicity, with many studies finding Black neonates are assigned significantly lower 1-min and 5-min Apgar scores than their White counterparts.^22,25,31–33^ Inconsistencies were still noted when only infants with normal blood gas measurements were included^31^ or when umbilical artery gases were statistically controlled for.^32^ Only the appearance component of the Apgar score differs by race, with Black neonates having significantly lower appearance scores even after controlling for multiple factors such as gestational age, cord gases, and maternal antenatal health.^32^ These scoring differences provide the most likely explanation of better survival among Black infants with low Apgar scores. In addition, differences in Apgar scoring are noted between hospitals, between professions such as neonatologists, midwives, and obstetricians,^34^ and between European countries with the proportion of neonates receiving an Apgar score of 10 varying from 9% to 93% in different countries.^35^ This suggests major differences in clinical training and convention when scoring the Apgar.^35^ These differences in assignment mean the significance of lower neonatal mortality among Black neonates with a low Apgar score is currently unclear. However, it may indicate that the appearance component which defines the highest score as “pink all over”, may not be a reliable component. Inconsistent results were also found in a few small studies regarding the ability of the Apgar score to predict long-term outcomes, however, this is generally considered outside the remit of the Apgar score.^36^

In clinical practice, although each neonate is assigned an Apgar score, it is suggested that it is seldom used to determine clinical management and is often assigned in retrospect.^37^ This is best demonstrated through a United States’s study which showed 90% of nurses assigned an Apgar score within a vignette even when data for some Apgar score components were absent, for example heart rate or respiration rate.^38^ A correct Apgar score was assigned only 19% to 57% of the time and inter-rater agreement was poor.^38^ This has additionally been confirmed through qualitative interviews, where healthcare professionals stated that they simply assigned an Apgar score of 9 or 10 if a neonate was alert and crying without actually assessing each component.^39^

While many high resource countries have moved away from using the Apgar score as a tool for decision-making and rely more on pulse oximetry and electrocardiograms, such medical technology is not always available especially in low resource settings and rural locations. It is therefore of paramount importance that more research is undertaken in countries where the Apgar score is still relied upon for clinical decision making for example some countries in Africa, Asian, and Latin America. Studies within these countries are also essential to better understand any inconsistencies in Apgar scoring where neonates with darker skin tones are in the majority. Additionally, despite questions around the reliability of the Apgar score for clinical decision making especially for neonates with darker skin pigmentation, its routine collection in practice means the Apgar score remains a focus of research studies. Within research there is an overemphasis on the Apgar score to classify neonatal wellbeing and to adjust within regression analyses, with little consideration of its reliability or validity.^37^ The limitations of the Apgar score, especially in those with darker skin tones needs to be more clearly understood within the research arena.

Replacements for the Apgar score have been proposed, for example, the Neonatal Resuscitation Assessment and Adaptation Score (NRAS)^40–43^ and the Expanded Apgar score which the American College of Obstetricians and Gynecologists recommend using if a neonate requires resuscitation.^36^ However, wider use of scores such as the NRAS is not currently recommended as it has only been assessed in small samples without explicit consideration of the predictiveness within neonates from different ethnicities.

It is known that visual changes to skin color with hypoxia may be less apparent in dark skinned neonates.^9,44^ In the absence of a pulse oximeter, hypoxia may not be detected in infants with darker skin tones by parents or professionals, resulting in later identification of deterioration. One small included study suggested tongue color was a good indicator of supplemental oxygen requirement in the delivery room, regardless of ethnicity.^26^ Generally however, visual assessment of cyanosis is poor, with pulse oximetry the mode of choice to detect cyanosis particularly in any neonatal resuscitation scenario.^10^ The COVID-19 pandemic highlighted inequalities in pulse oximetry reliability in Black, Asian or minority ethnic adults.^14,15,45^ One study included within this review also suggested pulse oximetry was less accurate in preterm neonates from Black and minority ethnic backgrounds, with slightly increased incidence of occult hypoxemia.^16^ Although pulse oximetry is better than visual assessment, it is suggested that saturations near the bottom of the recommended range are avoided in Black preterm neonates to minimize adverse outcomes.^16^ Studies in older children have shown mixed results. Two studies showed pulse oximetry overestimated arterial oxygenation more frequently in Black children.^46,47^ Occult hypoxemia was seen in 5.8% of White compared to 9.6% of Black children in one study^46^ and in 1% of White compared to 5% of Black children in the other study.^47^ In contrast another small study found no differences in pulse oximetry measurement bias between light and dark-skinned children when classifying children according to actual skin pigmentation, rather than ethnicity or race.^48^ A final study suggested that the absolute difference between pulse oximetry saturation and arterial saturation was lower in African American children, with a difference of more than 3% found in 30.0% of African American children compared to 48.9% of White children.^49^ Further research to examine the small but potentially clinically significant differences in pulse oximeter accuracy in neonates with diverse skin tones is warranted.

This review has highlighted the limitations of visual assessment of cyanosis especially among those with darker skin tones, as well as potential inconsistencies in scoring of the appearance component of the Apgar score. However, a recent review found that the skin color descriptors such as “pink”, “blue” and “pale” were still widely used within clinical guidelines and policies without consideration of how these may appear in neonates with different skin pigmentations.^50^ When concurrently considering that only 9.1% of staff report receiving adequate training around caring for ethnic minority babies,^24^ it highlights the urgent need to address care practices to ensure they are inclusive and safe for all communities that make up our diverse multiethnic society.

### Strengths and limitations

This review had several strengths. Firstly, a robust approach was applied including research of any methodology and scrutinizing literature by two reviewers. Secondly, a comprehensive search was undertaken, which included looking for gray literature.

Several limitations were however identified. Excluding non-English articles may have limited the number of studies available for inclusion. The international nature of included studies meant heterogeneity was noted in the categorization of ethnicity, race, or skin tone. Categories within the review retained the terms within the original articles, however, transferability of findings is complex. Additionally, all but one included study classified neonates according to ethnicity or race, rather than by skin tone. It is acknowledged that variations of skin tone occur within each race/ ethnic category, which are not captured within the included studies. Additionally, the heterogeneity in race/ ethnicity categories as well as in outcome measures meant that meta-analysis of the results was not possible. Included studies did not separate their results according to socioeconomic status, education level, and maternal age, therefore it was not possible to do a sub-group analysis according to these factors. The adequacy of provider training was not considered within the included studies that assessed neonatal outcomes; therefore it is unknown whether training, especially around assessment in dark skinned neonates may have impacted the results. Finally, only two included studies were deemed low risk of bias across all domains.

### Conclusions and implications

Low Apgar score and neonatal mortality were strongly correlated across all ethnicities, but variations in scoring practices, particularly affecting Black neonates, requires further attention. Additionally, further evaluation is needed to determine how to objectively assess newborn health. Visual detection of cyanosis is challenging, especially in neonates with darker skin, and therefore pulse oximetry is preferred to mitigate the health disadvantages experienced by those from ethnic minorities. However, small but potentially clinically significant differences in pulse oximetry compared to arterial oxygen saturation in neonates with darker skin tones warrants further exploration. Additionally, healthcare provider training gaps impact assessment accuracy. There is an urgent need for the development and robust prospective evaluation of targeted education around assessment in Black, Asian and minority ethnic neonates.

## Supplementary information

Appendix S1

PRISMA2020checklist

Supporting information Table S1

Supporting information Table S2

## Data Availability

All data generated or analyzed during this study are included in this published article and its online supplementary information files.
