# Supplementary material for: Systematic review of Apgar scores & cyanosis in Black, Asian, and ethnic minority infants
Source: Pediatr Res. 2024 Sep 14;97(3):939–52. doi: 10.1038/s41390-024-03543-3 (PMC12055595; doi:10.1038/s41390-024-03543-3)
Supplement: Supplementary file 1 — Appendix S1 [file 41390_2024_3543_MOESM1_ESM.pdf]

# Appendix S1: Example search strategy

## CINAHL

(MH "Anoxia") OR (MH "Apgar Score") OR (MH "Cyanosis") OR Hypoxia OR hypoxemia OR oximet* OR “oxygen saturation” OR jaundice OR APGAR OR cyanosis) OR ((MH "Jaundice") OR (MH "Jaundice, Neonatal") OR (MH "Hyperbilirubinemia, Neonatal") OR (MH "Hyperbilirubinemia")

AND (neonatal or newborn or neonate OR “new born” or new-born OR baby OR neonatol* OR Pediatric* OR paediatric* OR preterm OR premature OR babies)

AND ((MH "Ethnic Groups+") OR (MH "Minority Groups") OR (MH "Cultural Diversity") OR (MH "Asians+") OR (MH "Black Persons") OR (MH "Hispanic Americans") OR (MH "Indigenous Peoples") OR (MH "Immigrants+") OR (MH "Transients and Migrants") OR (MH "Emigration and Immigration") OR Black or Asian or “ethnic minority” or “minority ethnic” or bame or “Black or minority ethnic” OR bme OR “mixed race” OR “mixed ethnic*” OR “mixed heritage” OR skin pigmentation OR Hispanic OR Pakistan* OR Somali* OR India* OR Bangladesh* OR Chinese OR China OR Africa* OR Caribbean OR Arab OR Ethnic* OR ethno* OR race or Racial* or racism OR colour OR color) {LIMITED TO TITLE OR ABSTRACT OR SUBJECT}
