# Supplementary material for: Systematic review of Apgar scores & cyanosis in Black, Asian, and ethnic minority infants
Source: Pediatr Res. 2024 Sep 14;97(3):939–52. doi: 10.1038/s41390-024-03543-3 (PMC12055595; doi:10.1038/s41390-024-03543-3)
Supplement: Supplementary file 4 — Supporting information Table S2 [file 41390_2024_3543_MOESM4_ESM.pdf]

Table S2: Funding of included studies

Author	Study funding
**Apgar score studies**	
Chubb et al., (2022)	Not reported. Authors report no conflicts of interest
Gillette et al., (2022)	SJS is funded by a Wellcome Trust Clinical Career Development Fellowship 209560/Z/17/Z (https://wellcome.org). The funders had no role in study design, data collection and analysis, decision to publish, or preparation of the manuscript.
Li et al., (2013)	Ministry of Education of China; National Science Foundation of China; National Basic Research Program of China; Science and Technology Commission of Shanghai Municipality; Shanghai Municipal Education Commission; Shanghai Municipal Health Bureau; Morning Star Rewarding Fund of Shanghai Jiao Tong University; Xingbairen plan of Shanghai Jiao Tong University School of Medicine. Funders had no role in study design, data collection and analysis, decision to publish, or preparation of the manuscript
Mihoko Doyle et al., (2003)	National Institute of Child Health and Human Development
Serunian & Broman (1975)	National Institute of Neurological Diseases and Stroke contract to the Child Study Center, Brown University
Shankaran et al., (2004)	Supported by the National Institute of Child Health and Human Development
Wolf et al., (1997)	Not reported
**Cyanosis studies**	
Dawson et al., (2015)	JAD is a recipient of a National Health and Medical Research Council (NHMRC) Post Doctoral Fellowship (APP1012686) and is supported by the Victorian Government's Operational Infrastructure Support Program. COFK is a recipient of an NHMRC Early Career Fellowship. PGD is a recipient of an NHMRC Practitioner Fellowship. The study was supported in part by an NHMRC Program Grant No. 606789. CCR was supported by the European Respiratory Society through a 2011 Long Term Research Fellowship.
Goldman et al., (1973)	Not reported
Vesoulis et al., (2022)	NIH grant: K23 NS111086
